# Extrusion 3D Printing of Intrinsically Fluorescent Thermoplastic Polyimide: Revealing an Undisclosed Potential

**DOI:** 10.3390/polym16192798

**Published:** 2024-10-02

**Authors:** Premkumar Kothavade, Abdullah Kafi, Chaitali Dekiwadia, Viksit Kumar, Santhosh Babu Sukumaran, Kadhiravan Shanmuganathan, Stuart Bateman

**Affiliations:** 1 RMIT Centre for Additive Manufacturing, School of Engineering, RMIT University, Carlton, VIC 3053, Australia; premkumar.kothavade@rmit.edu.au (P.K.); abdullah.kafi@rmit.edu.au (A.K.); 2 Polymer Science and Engineering Division, CSIR-National Chemical Laboratory, Dr. Homi Bhabha Road, Pune 411008, Maharashtra, India; k.shanmuganathan@ncl.res.in; 3 Academy of Scientific and Innovative Research (AcSIR), Ghaziabad 201002, India; vk.yadav@ncl.res.in (V.K.); sb.sukumaran@ncl.res.in (S.B.S.); 4 RMIT Microscopy and Microanalysis Facility, STEM College, RMIT University, Melbourne, VIC 3000, Australia; chaitali.dekiwadia@rmit.edu.au; 5 Organic Chemistry Division, CSIR-National Chemical Laboratory, Dr. Homi Bhabha Road, Pune 411008, Maharashtra, India

**Keywords:** thermoplastic polyimide, additive manufacturing, high-performance polymers, functional extrusion 3D printing, fluorescent sensors, thermally stable polymers

## Abstract

Thermoplastic polyimides (TPIs) are promising lightweight materials for replacing metal components in aerospace, rocketry, and automotive industries. Key TPI attributes include low density, thermal stability, mechanical strength, inherent flame retardancy, and intrinsic fluorescence under UV light. The application of advanced manufacturing techniques, especially 3D printing, could significantly broaden the use of TPIs; however, challenges in melt-processing this class of polymer represent a barrier. This study explored the processability, 3D-printing and hence mechanical, and fluorescence properties of TPI coupons, demonstrating their suitability for advanced 3D-printing applications. Moreover, the study successfully 3D-printed a functional impeller for an overhead stirrer, effectively replacing its metallic counterpart. Defects were shown to be readily detectable under UV light. A thorough analysis of TPI processing examining its rheological, morphological, and thermal properties is presented. Extruded TPI filaments were 3D-printed into test coupons with different infill geometries to examine the effect of tool path on mechanical performance. The fluorescence properties of the 3D-printed TPI coupons were evaluated to highlight their potential to produce intricately shaped thermally stable, fluorescence-based sensors.

## 1. Introduction

High-performance polymers like polyetherimide (PEI), thermoplastic polyimides (TPI), and polysulfones (PSU) are recognised for their potential to replace metallic components in various industrial sectors, including transportation, marine, and electronic applications [1,2,3]. TPIs are known for their exceptional thermal and dimensional stability, inherent flame retardancy, and mechanical strength due to their polyimide structure. Whilst the chemical backbone of TPI provides advantages in mechanical and physical properties, it complicates the processing of these materials due to the high temperature required, high melt viscosity, and narrow processing window, necessitating specialised moulding equipment [4,5,6,7,8]. To enhance the processability of TPI, Gao et al. [9] undertook the preparation of blends combining TPI with PEEK. However, they encountered poor miscibility and subsequent phase separation in the TPI/PEEK blend. In a separate study, Nicholls et al. [10] introduced urea linkages into TPI to investigate the impact of additional hydrogen bonding. The resulting blend exhibited improved melt processability compared with fully aromatic PIs. In addition to this, significant research has been conducted on thermoplastic polyetherimides (PEI), such as those known under the tradename ULTEM (SABIC). Typically, ULTEM is synthesised through condensation polymerisation between bisphenol-A dianhydride (BPADA) and meta phenylene diamine (MPD) [11]. ULTEM 9085, a blend of PEI and polycarbonate (PC) copolymer [12], has been extensively researched in the realm of melt processing. The inclusion of PC enhances the melt processability of ULTEM 9085 but leads to trade-offs, such as a decrease in the overall physical properties of the blend relative to PEI [13]. Despite this and other strategies [14,15,16] investigated to enhance the processability of TPI without compromising inherent physical properties, achieving high-performance complex-shaped components remains a substantial challenge.

Additive manufacturing (AM), commonly referred to as 3D printing, represents an advanced manufacturing method employed to fabricate complex-shaped and customised objects in a time-efficient manner. Defined by the ISO/ASTM 52900:2021(E) standard, AM is the “process of joining materials to make parts from 3D model data, usually layer upon layer, as opposed to subtractive manufacturing and formative manufacturing methodologies.” [17]. It is categorised into seven distinct classifications. The advantages of 3D printing, such as cost-effectiveness, simplicity, and the ability to produce highly complex parts with minimal material usage, have positioned it as a preferred technique in the Industry 4.0 era [18,19,20,21]. While the 3D printing of commodity polymers like poly(lactic acid) (PLA), acrylonitrile butadiene styrene (ABS), nylon, polyethene terephthalate glycol (PETG), etc., are well-explored, the realm of 3D printing for high-performance polymers, particularly TPI, is still maturing [22,23,24,25]. This is mainly because of the challenges associated with high-temperature processing, such as high melt viscosity, a limited processing temperature range, the necessity for specialised equipment, and water absorption, among other factors. Among the various 3D-printing techniques, material extrusion and VAT polymerisation are the two categories explored for 3D printing of TPI polymer. Hegde et al. [26] achieved a milestone by demonstrating high-resolution 3D printing of pyromellitic dianhydride and 4,4′-oxydianiline (PMDA-ODA), commonly known as Kapton, using mask-projection stereolithography (MPSL) while preserving the polymers bulk properties. Despite the advancements, dimensional shrinkage limitations still existed. While VAT polymerisation techniques offer high accuracy, their high cost and the limited availability of photosensitive resins hinder widespread use [27,28]. Fused Deposition Modeling (FDM), or FFF (Fused Filament Fabrication), due to its low cost, simplicity, ease of operation, hardware availability, and solid raw material form, stands out as a widely used material extrusion process [29,30,31]. In a pioneering study on FDM 3D printing of TPI by Wu et al. [14], the authors explored the thermal stability of TPI pellets and filament, delving into the relationship between 3D-printing temperature and interlayer bonding force. Other studies, such as those by Ye et al. [15], have extended the exploration of FDM 3D printing of TPI to include investigations into the effects of carbon nanotubes (CNTs) on electrical conductivity, mechanical properties, cyclic bending deformation, and the impact of nozzle diameter variations. Additionally, they explored the influence of drying time on the tensile strength of 3D-printed TPI [16].

In the past few years, there has been tremendous growth in the 3D printing of functionalised materials for many advanced applications [32,33,34,35]. Amongst these functional properties, polyimides (PIs) have been discovered to possess intrinsic fluorescence properties [36,37,38]. Since the molecular structure of TPIs closely resembles that of PIs except for cross-linking, it was hypothesised that TPIs would also exhibit intrinsic fluorescence properties. This characteristic enhances the appeal of 3D printing TPIs for applications such as creating fluorescence-based complex-shaped sensors. In this study, we explored the processing of TPI filaments by examining rheology in relation to processing temperature and duration. The impact of different infill orientations (tool paths) during 3D printing on the mechanical properties of the TPI was also investigated, as were the thermal and fluorescence characteristics of the 3D-printed TPI. For a practical demonstration, we 3D-printed a functional impeller for an overhead stirrer, effectively replacing its metallic counterpart and enabling easy defect detection under UV light. To our knowledge, this is the first study to successfully extrude TPI filaments, explore 3D printing with various infill directions to understand their mechanical properties and investigate fluorescence properties. Moreover, we demonstrated the creation of a functional product capable of replacing a metallic equivalent. These 3D-printed TPI components could be ideal for applications requiring high-temperature stability, intricate shapes, and fluorescence properties.

## 2. Experimental Section

### 2.1. Materials

Thermoplastic polyimide (TPI) (EXTEM RESIN VH1003), having a density of 1.3 g/cm^3^ and a melt flow rate (MFR) of 15.5 g/10 min (measured at 367 °C at 6.6 kgf), was procured from Nagase Singapore (Pte) Ltd., Wheelers Hill VIC, Australia branch office, and supplied by SABIC, Clayton VIC, Australia. The glass transition temperature (T_g_) and melting temperature (T_m_) of TPI were in the ranges of 245–250 °C and 380–405 °C, respectively. The heat deflection temperature (HDT) of TPI was in the range of 235–240 °C at 0.45 MPa. Polyetherimide (PEI) film with a 500-micron thickness (3M 468MP 200MP Adhesive) was procured from Aurarum, Ringwood VIC, Australia, and supplied by 3M, Glen Waverley VIC, Australia. A nano polymer adhesive for a high-temperature build plate was procured from Vision Miner, Santa Ana CA, USA.

### 2.2. Preparation of TPI Filaments

#### 2.2.1. Pellet Drying

Prior to filament extrusion, TPI pellets were dried in an oven at 150 °C for 4–6 h under a continuous vacuum. The moisture absorption test was carried out after drying, and it was ensured that the moisture level was less than 200 ppm to avoid any processing challenge due to entrapped moisture.

#### 2.2.2. Extrusion Process

The dried TPI pellets were subjected to melt extrusion in a micro-scale single screw extruder (Model: Xcalibur by Noztek, Shoreham-by-Sea, England) with three temperature zones (420 °C, 410 °C, and 395 °C) at 20 rpm and a residence time of less than 4 min. The extruder was equipped with a full filament extrusion line, including a water bath, tolerance puller and filament winder. As soon as the hot TPI extrudate exited the die, it was passed through a cold-water bath and taken up to the filament winder through a tolerance puller. The processing conditions, screw speed, and pulling speed of the tolerance puller were optimised to obtain a 1.75 ± 0.05 mm diameter. The diameter of the filament was further verified by a vernier caliper at multiple locations.

### 2.3. FDM 3D Printing of TPI Filaments

Extruded filaments were dried in a vacuum oven at 150 °C for 4–6 h before 3D printing. All test specimens were printed using a custom-built high-temperature WOMBOT FDM 3D printer utilising BONDTECH printheads with a 0.6 mm diameter hardened steel nozzle. Cura 5.4.0 slicer software was employed with the surface tessellation language (STL) file for the tensile testing specimen (ASTM D638-Type1) and films for fluorescence characterisation designed using Autodesk Fusion 360 software. The mechanical properties of the FDM-printed coupons vary when printed with different infill directions, such as 0°, 45°, and 90° [39,40]. Therefore, test specimens were printed using all three infill directions to better understand the impact of infill orientation on the mechanical properties of 3D-printed TPI. Parameters like printing speed, infill density, layer height, and infill pattern were optimised by multiple iterations to achieve ‘fully dense’ solid 3D-printed objects. Non-adherence of TPI to the glass bed was observed after the first few prints, and to tackle this challenge, a PEI bed was used as a build surface, along with the application of nano polymer adhesive. Table 1 outlines the optimised parameters for 3D printing the prepared TPI filaments, determined through a trial-and-error method.

### 2.4. Characterisation Methods

#### 2.4.1. Moisture Analysis

The moisture content of the TPI pellets was analysed using the Moisture Analyzer (Model: HR83 by Mettler Toledo, Melbourne VIC, Australia). TPI pellets of 40–50 g were used for the analysis. TPI pellets were evenly spread on an aluminium pan, and a standard drying program was employed, in which a drying temperature of 150 °C and timed switch-off criteria of 300 min were implemented. At the end of the test, moisture content was calculated according to the principle of thermogravimetry and reported as a percentage (%).

#### 2.4.2. Rheological Characterisations

Dried TPI pellets (4–6 h at 150 °C) were used to prepare compression-moulded discs with a 25 mm diameter and a 2.5 mm thickness for rheological characterisations. A parallel plate geometry was used for performing rotational rheology experiments on a stress-controlled shear rheometer (Model: Discovery HR3 Hybrid Rheometer by TA Instruments, New Castle, DE, USA). The hot-pressed discs were dried for 4–6 h at 150 °C before being tested. All measurements were performed for 20 min under a nitrogen atmosphere to prevent thermo-oxidative degradation.

#### 2.4.3. Mechanical Characterisations

TPI specimens were 3D-printed in three infill directions—0°, 45°, and 90°—and tested according to ASTM D 638 on an universal testing machine (UTM) (Model: Instron 5900R 4467, Norwood, MA, USA). A load cell of 5 kN and a strain rate of 5 mm/min were used. The tensile strength, elongation at break, and tensile (or Young’s) modulus were measured. Testing was performed under ambient conditions, and the data reported are the average of a minimum of five readings along with the standard deviation. Toughness was calculated as the integrated area under the tensile stress–strain curve using Origin9 data analysis and graphing software. The trapezoidal rule method was used to calculate the area under the curve.

#### 2.4.4. Fluorescence Measurements

Emission and excitation spectra were performed using PTI Quanta Master™ Steady State Spectrofluorometer by Horiba Instruments, Edison, NJ, USA. Fluorescence lifetimes were measured by time-correlated single-photon counting (TCSPC) using a spectrofluorometer, and the LED excitation source was 374 nm. The quality of the fit was judged by fitting parameters such as *χ*^2^ (<1.25) and visual inspection of the residuals. Photoluminescence quantum yield was measured using a Fluoromax plus spectrofluorometer_QY absolute PL quantum yield spectrometer. Fluorescence characterisations were performed on 3D-printed films of all compositions. The dimensions of the 3D-printed films in a square shape were 30 × 30 × 0.3 mm (l × w × h).

#### 2.4.5. Differential Scanning Calorimetry and Thermogravimetric Analysis

Differential scanning calorimetry (DSC) (TA instruments DSC250, New Castle, DE, USA) and a simultaneous thermal analyser (STA) (NETZSCH Jupiter 449 F5, Selb, Germany) were used to ascertain thermal behaviour. The thermal behaviour of TPI pellets, extruded TPI filaments, and 3D-printed TPI samples was investigated using 7–8 mg of each in STA and DSC measurements. STA was performed from 40 °C to 900 °C using a 10 °C/min heating rate under a N_2_ atmosphere. For DSC, samples were heated from 30 °C to 400 °C at 10 °C/min, kept isothermal for 5 min, cooled to 30 °C at 10 °C/min, and heated again to 400 °C.

#### 2.4.6. Field-Emission Scanning Electron Microscopy

Field-emission scanning electron microscopy (FE-SEM) (Quanta 200 by FEI, Oregon, USA) was used at an accelerating voltage of 30 kV to study the surface morphology, cross-sectional area, and fracture mechanics of the 3D-printed TPI specimens. The samples were subjected to a thin coating of iridium under vacuum before imaging. The cross-section of fractured specimens during tensile testing was used to study morphology using FE-SEM.

#### 2.4.7. X-Ray Micro-Computed Tomography Imaging

The cross-section of fractured specimens during tensile testing was used to study morphology using a 3D X-ray Micro-Computed Tomography imaging (MicroCT) system (Model: Skyscan 1275 by Bruker, Preston VIC, Australia). MicroCT was used to examine the packing of the 3D-printed TPI. Overall projection images were obtained at 40 kV with no filter at 0.5 degrees of rotation at 360 degrees. The scanned data were reconstructed using NRecon and rendered in CTVox software. The porosity was calculated using a CT analyser (CTan) for datasets in 2D and 3D for morphometry and densitometry.

## 3. Results and Discussion

### 3.1. Moisture Content Analysis and Processing of TPI for Filament Production

**Figure 1:**
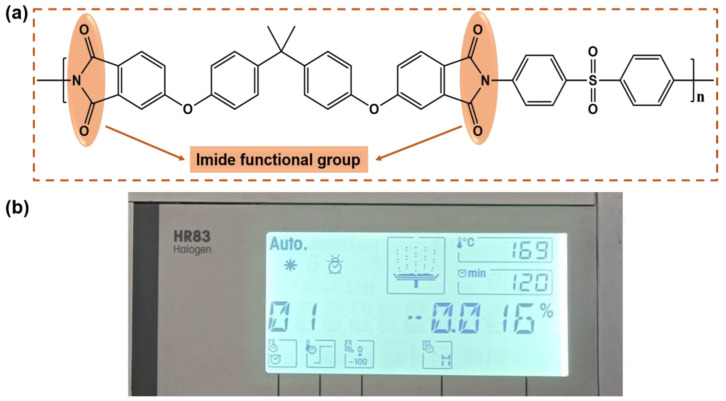
(**a**) Molecular structure of TPI wherein polar imide group is highlighted. (**b**) Moisture analyser program.

The molecular structure of TPI is illustrated in Figure 1a, and it is widely acknowledged that the presence of the highly polar imide functional group in its chemical composition makes TPI prone to rapid moisture absorption through hydrogen bonding or other intermolecular forces. This characteristic presents notable challenges during processing, such as the formation of bubbles and an uneven surface due to bubble bursts at processing temperatures. Additionally, the entrapment of moisture can significantly compromise or deteriorate various properties of melt-processed TPI, including the dimensional accuracy of the printed part, mechanical strength, and electrical properties. Such extruded TPI filaments, consequently, are not recommended for 3D printing. Ye et al. [16] conducted a study highlighting that TPI, when well-dried, demonstrates improved mechanical properties attributed to reduced porosity and enhanced processability. Hence, it is crucial to ensure that the moisture content in TPI pellets before melt processing falls within the recommended limit of <200 ppm [41]. To achieve this, TPI pellets were dried at 150 °C for 4–6 h under continuous vacuum before melt processing. The moisture content of these dried TPI pellets was then analysed, revealing that drying at 150 °C for 4–6 h effectively removes absorbed moisture. Notably, our observations indicate that even a 2 h drying period at 170 °C under the specified conditions is adequate to reduce the moisture level below 200 ppm (Figure 1b).

**Figure 2:**
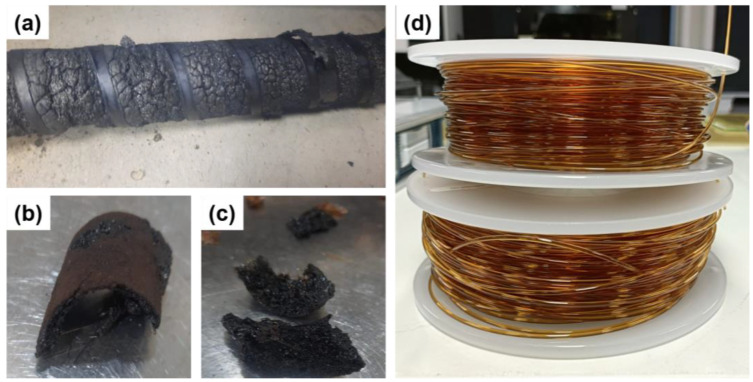
(**a**–**c**) Degraded TPI polymer due to the prolonged residence time. (**d**) Extruded TPI filament.

TPI is renowned for its high T_g_ and robust thermal stability, attributes stemming from its rigid aromatic backbone. Nevertheless, this inherent rigidity presents significant hurdles during the melt processing of TPI, giving rise to issues such as high viscosity and an extremely limited processing temperature range. The high viscosity and prolonged residence time complicate extrusion operation, often leading to polymer melt degradation (Figure 2a–c). Various solutions have been proposed to address these challenges, such as modifying the screw design, incorporating flexible groups during TPI synthesis, and introducing processing additives. However, these remedies often prove costly and time-consuming and alter the fundamental properties of TPI. In this study, we demonstrated the processability of TPI by solely optimising key process parameters, including nozzle and barrel temperature, residence time, and screw speed. The optimisation of these parameters was achieved through comprehensive studies elucidated in subsequent sections, encompassing analyses of viscosity changes over time at different temperatures and thermal and morphological characterisations. Our findings indicate that extrusion at 420 °C, with a residence time of less than 4 min at 20 rpm, represents the optimised process conditions for obtaining high-quality TPI filament. Importantly, we illustrated the scalability of this process (Figure 2d). A movie showcasing the complete TPI filament extrusion line can be accessed in the Supplementary Information (Movie S1).

### 3.2. Rheological Characterisation

**Figure 3:**
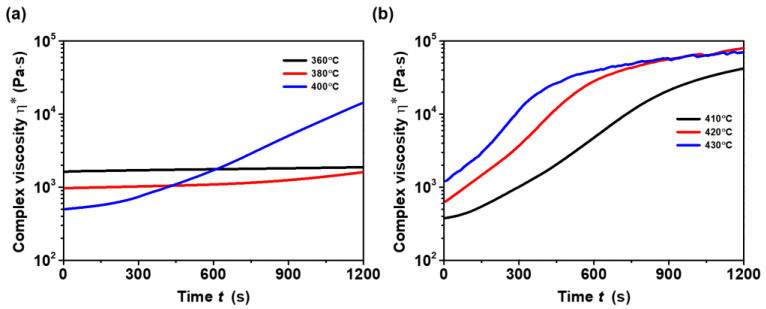
Complex viscosity-time curves of TPI at (**a**) 360, 380, and 400 °C and (**b**) 410, 420, and 430 °C.

Figure 3a illustrates changes in complex viscosity with respect to time. At lower temperatures of 360 °C and 380 °C, the complex viscosity of the TPI remained stable with respect to time (up to 20 min). However, at these temperatures, the melt extrusion process was notably inadequate, and at 360 °C, the barrel became jammed, and the screw stopped, which can be linked to the improper melting of the TPI. Similar outcomes were noted during TPI extrusion at 380 °C. The extruded TPI at 380 °C and 400 °C displayed irregular diameters and partially melted TPI pellets post-extrusion.

At temperatures 400 °C and above, complex viscosity increased gradually with an increase in temperature (Figure 3b). As previously noted, even when TPI was extruded at 400 °C, it did not exhibit satisfactory melt flow, resulting in filaments with uneven diameters. At temperatures of 410 °C, 420 °C, and 430 °C, complex viscosity increased rapidly over time. The rise in the complex viscosity of the TPI with time at higher processing temperatures can be attributed to a variety of factors or a combination thereof. Several authors have documented that thermoplastic polymers may experience an increase in molecular weight over time through processes such as chain extension, branching, or cross-linking [42,43,44]. These reactions may occur due to heat, radiation, and chemical agent exposure, resulting in longer polymer chains and increased entanglement. Consequently, the polymer’s resistance to flow, as reflected in its complex viscosity, tends to increase. It is widely acknowledged that all PIs display robust intermolecular interactions [45,46]. Hence, the increase in complex viscosity is also likely tied to the gradual structuring of TPI’s macromolecules over time. Filaments obtained at 410–430 °C temperatures had excellent melt flow and were of uniform diameter. By considering all these factors, a processing window of 410–430 °C was chosen, and all filaments used in this study were processed in this processing window.

### 3.3. 3D Printing of TPI in Different Infill Directions and Their Mechanical Characterisation

**Figure 4:**
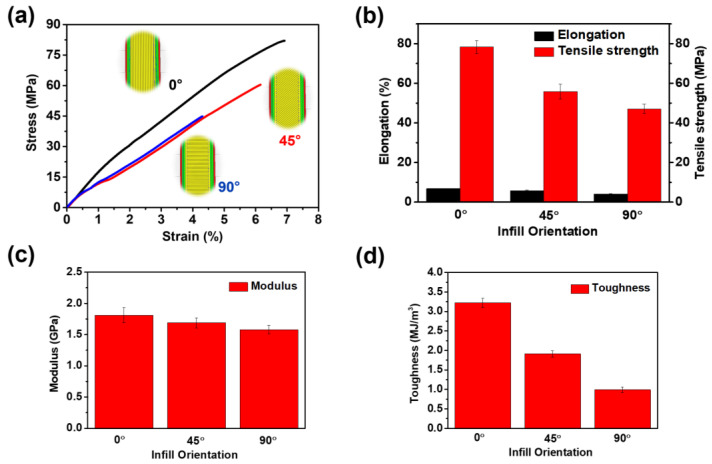
(**a**) Tensile stress–strain curves for TPI printed in different infill directions. (**b**) Measured tensile strength and elongation at break. (**c**) Young’s modulus and (**d**) calculated tensile toughness values.

It is well known that 3D printed products consistently demonstrate inferior mechanical properties when compared to moulded products [47,48]. This discrepancy is primarily attributed to the weaker interlayer adhesion present in 3D printing processes in contrast to products manufactured through high-pressure moulding techniques such as injection moulding and compression moulding. The layer-by-layer construction in 3D printing can lead to reduced bonding strength between layers, impacting the overall mechanical performance of the printed objects. In contrast, moulding techniques exert high pressure, facilitating stronger material cohesion and enhanced mechanical properties in the final moulded products. Moreover, 3D printed products exhibit anisotropic properties based on tool path and interlayer bonding. Anisotropic properties imply that the mechanical strength and behaviour of the material can vary significantly depending on the direction in which it is tested or applied. Therefore, it is crucial to investigate the mechanical characteristics of TPI when 3D printed in different infill directions, including 0, 45, and 90°, as its study provides a comprehensive understanding of its performance under varied conditions, essential for optimising its use in specific applications. As shown in Figure S1, dogbone-shaped tensile specimens of TPI were 3D printed in the 0, 45, and 90° infill directions using the printing parameters listed in Table 1. A video of the FDM 3D printing of prepared TPI filament can be accessed in the Supplementary Information (Movie S2). The printed specimens underwent testing on a UTM, and the results for tensile strength, tensile modulus, elongation at break, and calculated tensile toughness are illustrated in Figure 4 and Table S1.

According to EXTEM^TM^ TPI’s technical data sheet, moulded TPI displays a tensile strength of 96 MPa, elongation at break of 6%, and a tensile modulus of 3.51 GPa [49]. Whilst specimens 3D printed with a 0° infill orientation exhibited the highest mechanical properties (compared with 45° and 90° orientations), mechanical testing revealed a tensile strength of 78 MPa, an elongation at break of 6.8%, and a tensile modulus of 1.81 GPa, an 18% and 48% reduction in strength and stiffness respectively. This decline in mechanical properties is likely attributable to porosity and weaker fusion between printed layers in 3D printed coupons in contrast to those manufactured by moulding. Comparing coupons manufactured with different raster orientations, the reduction in mechanical properties observed at 45° and 90° relative to 0° presumably results from loading out of plane to the raster. With the 0° infill orientation, the load is primarily carried by the polymer rasters themselves, whereas in the 90° infill orientation, the load is borne by interfacial bonds between the layers [39]. Failure in this former (0°) orientation requires breaking the rasters and necessitates high stress due to the high levels of polymer chain entanglement and lower potential for porosity. As the infill angle increases from 0 to 90, a larger proportion of stress is applied perpendicular to the rasters, leading to failure through raster delamination since the degree of polymer entanglement (hence adhesion) between layers is not as well developed due to cooling effects. Since the tensile strength between rasters is significantly lower, failure occurs at lower stresses [40]. Morphological characterisations confirmed that in the 0° infill orientation, failure occurred through raster rupture, while in the 90° infill orientation, failure was due to clear separation between printed layers or interfacial separation. To the best of our knowledge, this study is the first to calculate the tensile toughness of 3D-printed TPI by determining the area under the stress–strain curve. The resulting values were 3.22 MJ/m^3^, 1.91 MJ/m^3^, and 0.99 MJ/m^3^ for samples produced with 0°, 45°, and 90° infill directions, respectively. These findings suggest the potential for enhancing the mechanical properties of 3D-printed TPI to match those of moulded specimens through the synergistic effect of the incorporation of various reinforcements and the optimisation of processing parameters, including infill direction.

### 3.4. Fluorescence Characterisation

**Figure 5:**
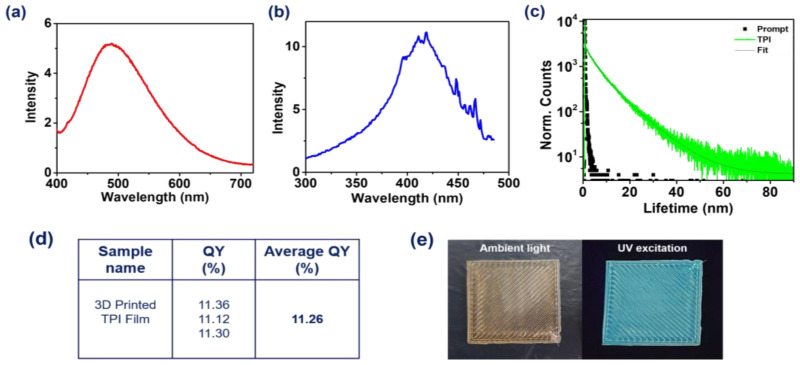
(**a**) Fluorescence (*λ*_ex_ = 411 nm), and (**b**) excitation spectra (*λ*_ex_ = 490 nm), along with (**c**) fluorescence lifetime decay profile (*λ*_mon_ = 490 nm, *λ*_ex_ = 374 nm) of 3D-printed polymer films at 25 °C. (**d**) Fluorescence quantum yield and (**e**) photographs of the 3D-printed TPI films under ambient light (left) and UV light (365 nm) (right).

The fluorescence spectra of 3D-printed TPI films measured by exciting at a 411 nm wavelength show a broad spectrum in the range of 400–700 nm (Figure 5a), with a fluorescence maximum of 490 nm. In addition, the excitation spectrum of 3D-printed TPI shows a broad peak spanning 325–475 nm (Figure 5b), hinting at the absorption features. The fluorescence lifetime of 3D-printed TPI film was found to be 9.8 ns (53.93%) when monitored at the fluorescence maxima (*λ*_mon_ = 490 nm) (Figure 5c). Moreover, the average fluorescence quantum yield of the 3D-printed TPI film was 11.26%. This value is in agreement with the results reported by Zhou et al. [38] for wholly aromatic solution-prepared films. According to our knowledge, this is the first report in which 3D-printed TPI films were studied for their fluorescence properties. TPI films were 3D printed in rectangular shapes with dimensions of 30 × 30 × 0.3 mm (l × w × h) for fluorescence characterisation, and the corresponding photographs were taken under visible as well as UV light (Figure 5e); 3D-printed TPI film exhibited light blue fluorescence under UV light (365 nm).

### 3.5. Morphological Characterisation

**Figure 6:**
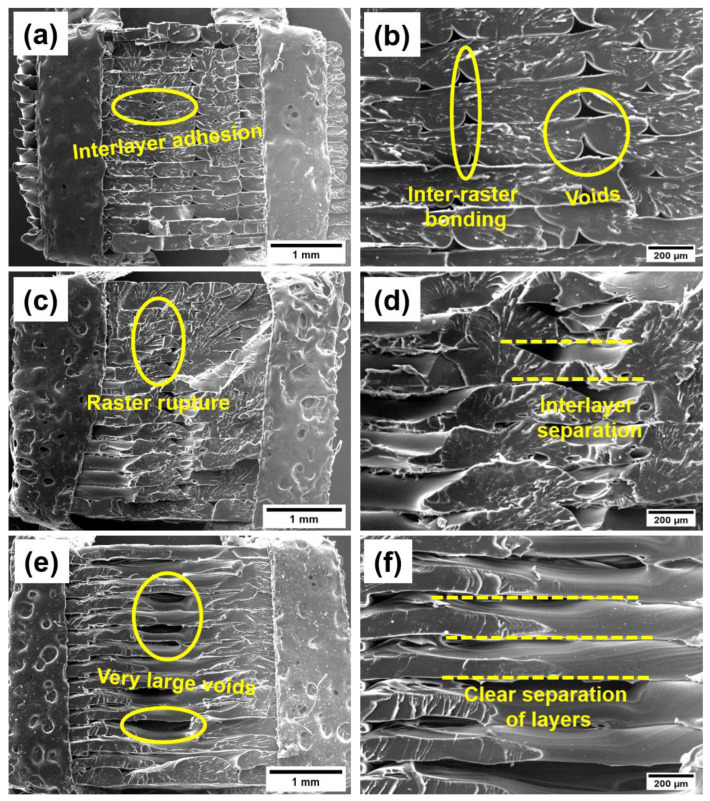
SEM micrographs of TPI 3D printed in (**a**,**b**) 0°, (**c**,**d**) 45°, and (**e**,**f**) 90° infill directions.

**Figure 7:**
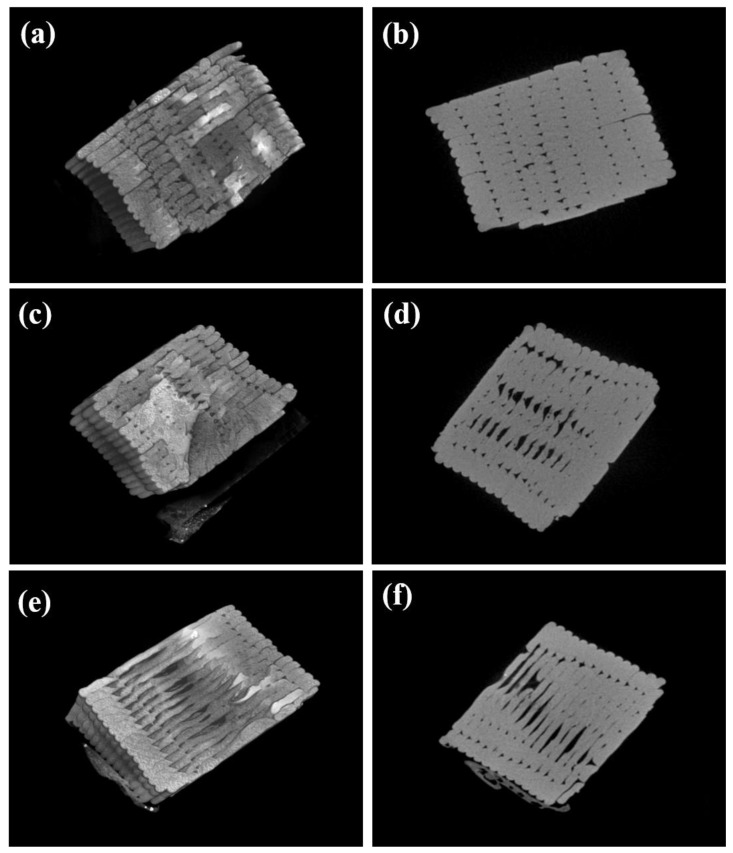
X-ray microtomography 3D images (**a**,**c**,**e**) and 2D slices (**b**,**d**,**f**) of tensile fractured 3D-printed TPI in (**a**,**b**) 0°, (**c**,**d**) 45°, and (**e**,**f**) 90° infill direction.

To elucidate the impact of infill directions on the mechanical properties of the 3D-printed TPI, the tensile fractured cross-sectional areas of the TPI specimens printed with 0°, 45°, and 90° infill orientations using SEM and Micro-CT imaging were examined. Due to the layer-by-layer nature of 3D printing, voids and weld points are inherent in the samples, resulting in a notable reduction in strength compared with their moulded counterparts. Figure 6a,b and Figure 7a,b illustrate that TPI printed with a 0° infill exhibits stronger interlayer and inter-raster adhesion, with minimal voids between layers and rasters. Conversely, specimens printed with a 90° infill (Figure 6e,f and Figure 7e,f) show a clear separation between layers and the presence of large voids, explaining their inferior mechanical properties. The microscopic images indicate that while the rasters carry the load in samples produced with a 0° infill direction, the load is carried by interfacial bonding in samples with a 90° infill direction. For specimens printed with a 45° infill, the damage mechanism involves both interface separation and raster breaking (Figure 6c,d and Figure 7c,d). Microscopic images of the fracture surfaces corroborate these findings, revealing adhesion and delamination between rasters and layers across different infill directions. The porosity values were determined using morphometry analysis from micro-CT data. The CTan performed data analysis on the basis of the white and black pixels to determine pores in the samples. The calculated total porosity for 0, 45, and 90° infill were 12, 16, and 24%, respectively. It was found that 0° infill exhibited the least amount of porosity compared with 90° infill, which correlates well with the observed mechanical properties.

### 3.6. Thermal Characterisation

**Figure 8:**
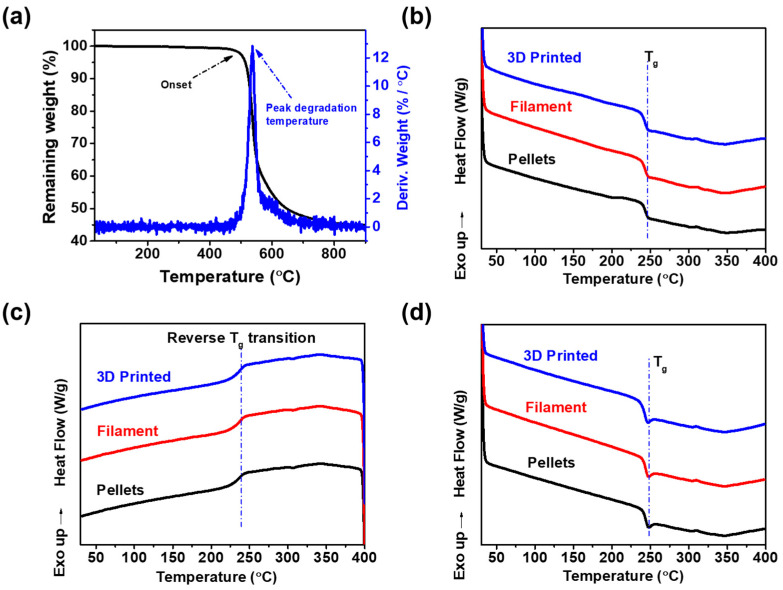
(**a**) TGA thermogram of 3D-printed TPI; (**b**) first heating scans; (**c**) cooling scans; and (**d**) second heating scans of TPI pellets, extruded filament, and 3D-printed TPI.

The results from the thermogravimetric analysis (TGA) between (50 to 900 °C) disclosed that the 3D-printed TPI exhibited a single onset degradation temperature at 514 °C with 5% weight loss, and the peak degradation temperature occurred at 538 °C. To assess the impact of processing on the thermal properties of 3D-printed TPI, TGA was conducted on all three samples, namely, as supplied TPI pellets, extruded filaments, and the 3D-printed TPI. Notably, no significant changes were observed in the onset degradation and peak decomposition temperatures. This contrasts with the findings of Wu et al. [14], who reported a 5.52% reduction in the onset degradation temperature of TPI following extrusion melt processing. The TGA thermogram and its derivative curve for 3D-printed TPI are depicted in Figure 8a, while those for the as-supplied pellets and extruded filaments can be seen in Figures S2 and S3, respectively.

DSC heating scans revealed the endothermic transitions in the range of 243–247 °C, which depicted the T_g_ of TPI (Figure 8b,d). This T_g_ value aligns closely with the T_g_ value reported elsewhere [49]. No indications of cold crystallisation (T_cc_) or crystallisation (T_c_) were observed during the heating and cooling scans, respectively. Similarly, during the cooling scans, an exothermic response in the same temperature range of 243–247 °C was noted, providing additional confirmation of the T_g_ of TPI and affirming the material’s stability and capability to maintain its amorphous state (Figure 8c). Contrary to a sharp melting point, the DSC heating scans showcased a gradual, broadening change in heat capacity, indicative of the amorphous nature of the TPI polymer. As observed in the DSC results, there were no significant differences noted in the T_g_ among TPI pellets, extruded filaments, and 3D-printed TPI. These findings from thermal characterisations indicate that the fundamental thermal properties of TPI remain consistent even after undergoing thermal processing and 3D printing. The consistent onset degradation, peak decomposition, and T_g_ values observed across various forms of TPI (pellets, filaments, and 3D-printed structures) suggest the preservation of the material’s amorphous structure, underscoring its integrity and relevance for its intended performance and applications.

### 3.7. 3D-Printed Functional Impeller

**Figure 9:**
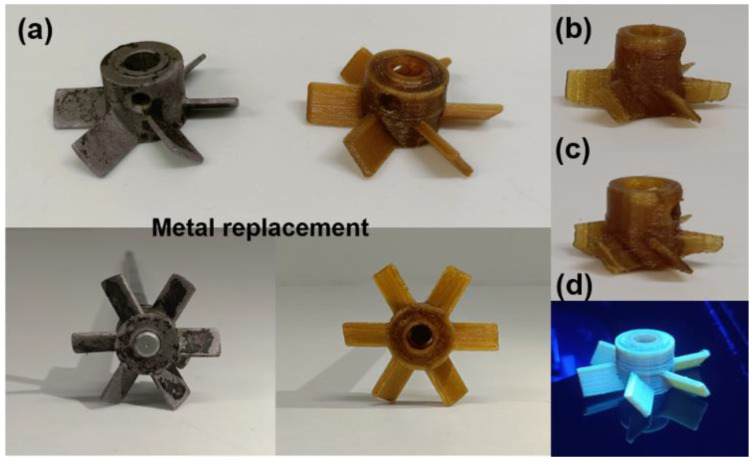
(**a**) TPI 3D-printed impeller as a replacement for existing metallic impeller. (**b**,**c**) Customised impellers. (**d**) Picture of printed impeller under UV light (365 nm).

To demonstrate the practical applicability of this processed inherent fluorescent TPI, we 3D printed an impeller for an overhead stirrer. This impeller successfully replaced its mechanical counterpart (Figure 9a). A demonstration of the overhead stirrer operating with the TPI 3D-printed impeller can be viewed in Movie S3. Furthermore, we 3D-printed a small-sized impeller (Figure 9b) and another with sharp blades (Figure 9c) to demonstrate the customisation potential for specific applications. Images of the functional impeller were taken under UV light to emphasise its fluorescent properties. This imaging technique also allows for the detection of even minor printing defects in parts made from this material (Figure 9d). This opens up new application windows for this material in situations that demand high-temperature stability, intricate shapes, and fluorescence properties as essential requirements.

## 4. Conclusions

In summary, this research highlights the considerable promise of TPIs as adaptable materials suitable for advanced applications, especially in sectors that demand lightweight, thermally stable, and mechanically resilient components. The inherent fluorescence of TPIs, with a measured solid-state quantum yield of 11.26%, adds a fascinating aspect to their versatility. Despite the existing challenges in processing, this research successfully addresses the limitations by delving into the production of TPI filaments and their subsequent 3D printing in different infill directions. The comprehensive examination of rheological, morphological, and thermal properties contributes to a deeper understanding of TPI processing. The 3D printing and mechanical analysis in different infill directions further substantiate their viability for practical applications. Of all the specimens, those printed with a 0° infill orientation exhibited the highest mechanical properties, achieving a tensile toughness of 3.22 MJ/m^3^. Moreover, the revelation of fluorescence properties introduces a novel aspect, positioning 3D-printed TPI as a potential material for constructing thermally stable, intricately shaped fluorescence-based sensors. This pioneering study marks the first demonstration of simultaneous TPI processing for filament production, 3D printing in various orientations, and fluorescence characterisation. It includes the fabrication of a functional impeller for an overhead stirrer, which has the potential to replace its metallic counterpart. The 3D-printed fluorescent TPI not only expands the horizon of material possibilities but also opens avenues for the development of sophisticated, uniquely shaped sensors with diverse applications in different sectors. These results highlight the potential of TPI for not only replacing metal parts in high-temperature applications but also in developing advanced fluorescent sensors for industrial uses. In essence, this study underscores the transformative impact of combining TPIs, 3D printing, and fluorescence properties, offering a promising direction for future research and practical implementations in cutting-edge technological domains.

## Figures and Tables

**Table 1:** Parameters used for FDM 3D printing.

Parameter	Value
Printing temperature	420 °C
Printing speed	40 mm/s
Bed temperature	130 °C
Cooling	0%
Infill density	100%
Infill pattern	Lines
Layer height	mm

## Data Availability

All data are contained within this article and the Supplementary Material provided.

## Supplementary Materials

The following supporting information can be downloaded at: https://www.mdpi.com/article/10.3390/polym16192798/s1, Figure S1: The 3D-printed TPI tensile specimens in 0, 45, and 90° infill directions; Figure S2: TGA thermogram and its derivative curve of TPI pellets; Figure S3: TGA thermogram and its derivative curve of extruded TPI filaments; Table S1: Tensile strength, tensile modulus, elongation at break, and calculated tensile toughness of 3D-printed TPI in 0, 45, and 90° infill directions; Movie S1: A complete TPI filament extrusion process; Movie S2: FDM 3D printing of prepared TPI filament; Movie S3: The 3D-printed TPI impeller in operation.
