# Supplementary material for: Extrusion 3D Printing of Intrinsically Fluorescent Thermoplastic Polyimide: Revealing an Undisclosed Potential
Source: Polymers (Basel). 2024 Oct 2;16(19):2798. doi: 10.3390/polym16192798 (PMC11478566; doi:10.3390/polym16192798)
Supplement: Supplementary file 1 [file polymers-16-02798-s001.zip › polymers-3203344-supplementary.pdf]

polymers

Supporting Information

Extrusion 3D Printing of Intrinsically Fluorescent Thermoplastic Polyimide: Revealing an Undisclosed Potential

Premkumar Kothavade [1,2,3], Abdullah Kafi [1], Chaitali Dekiwadia [4], Viksit Kumar [3,5], Santhosh Babu Sukumaran [3,5], Kadhiravan Shanmuganathan [2,3] and Stuart Bateman [1,*]

1 RMIT Centre for Additive Manufacturing, School of Engineering, RMIT University, Carlton, VIC 3053, Australia; premkumar.kothavade@rmit.edu.au (P.K.); abdullah.kafi@rmit.edu.au (A.K.)
2 Polymer Science and Engineering Division, CSIR-National Chemical Laboratory, Dr. Homi Bhabha Road, Pune 411008, Maharashtra, India; k.shanmuganathan@ncl.res.in
3 Academy of Scientific and Innovative Research (AcSIR), Ghaziabad 201002, India
4 RMIT Microscopy and Microanalysis Facility, STEM College, RMIT University, Melbourne, VIC 3000, Australia; chaitali.dekiwadia@rmit.edu.au
5 Organic Chemistry Division, CSIR-National Chemical Laboratory, Dr. Homi Bhabha Road, Pune 411008, Maharashtra, India; vk.yadav@ncl.res.in (V.K.); sb.sukumaran@ncl.res.in (S.B.S.)
* Correspondence: stuart.bateman@rmit.edu.au

The PDF file includes:

Figure S1. 3D printed TPI tensile specimens in 0, 45, and 90° infill directions
Figure S2. TGA thermogram and its derivative curve of TPI pellets
Figure S3. TGA thermogram and its derivative curve of extruded TPI filaments
Table S1. Tensile strength, tensile modulus, elongation at break, and calculated tensile toughness of 3D printed TPI in 0, 45, and 90° infill directions

Other Supplementary material for this manuscript includes the following:

Movie S1. A complete TPI filament extrusion process
Movie S2. FDM 3D printing of prepared TPI filament
Movie S3. 3D printed TPI impeller in working

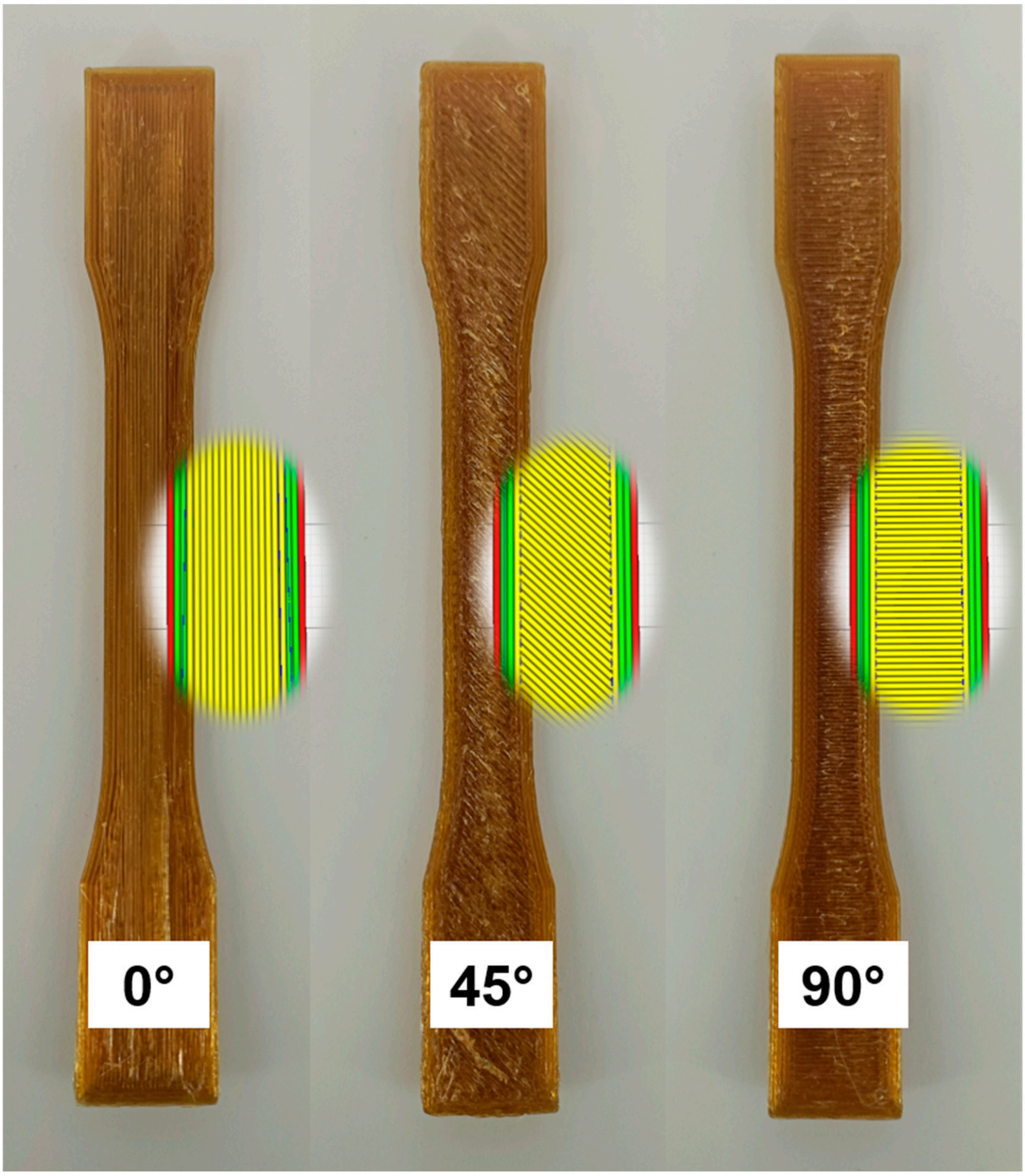

Figure S1. 3D printed TPI tensile specimens in 0, 45, and 90° infill directions.

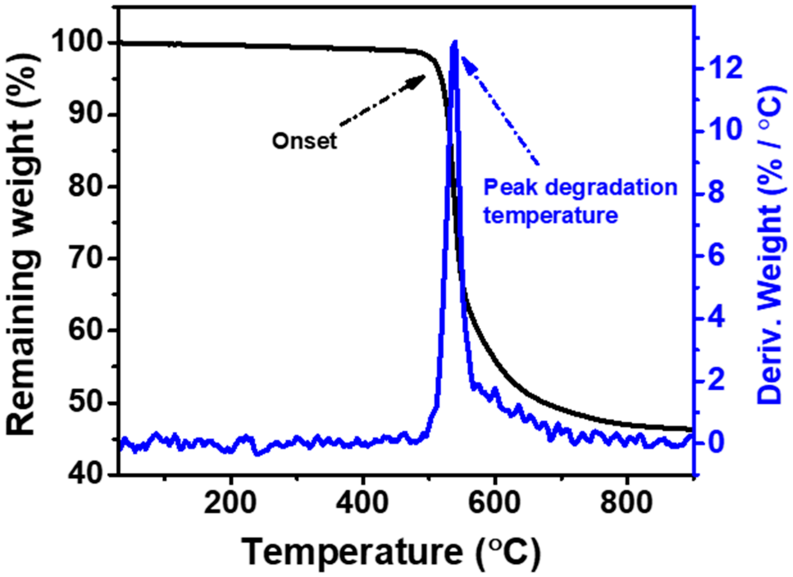

Figure S2. TGA thermogram and its derivative curve of TPI pellets.

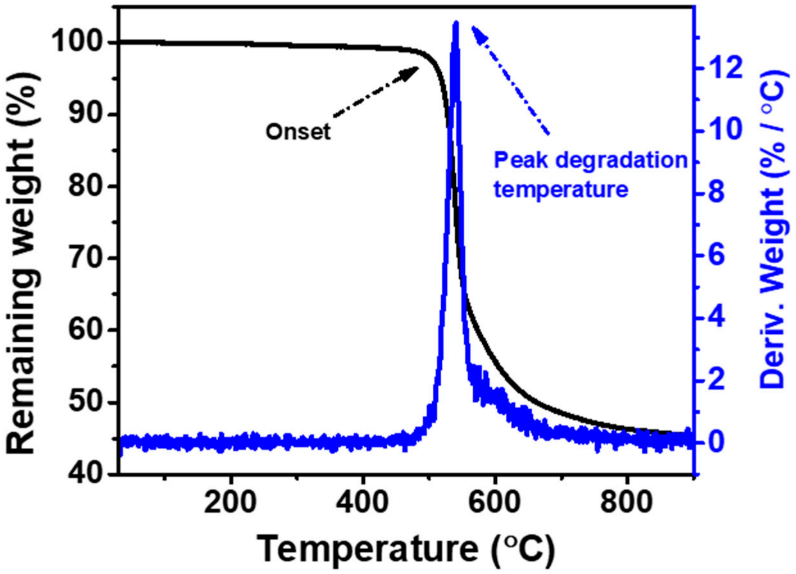

Figure S3. TGA thermogram and its derivative curve of extruded TPI filaments.

Table S1. Tensile strength, tensile modulus, elongation at break, and calculated tensile toughness of 3D printed TPI in 0, 45, and 90° infill directions.

3D printed TPI Specimens	Tensile Strength (MPa)	Elongation at Break (%)	Tensile Modulus (GPa)	Tensile Toughness (MJ/m³)
0 °	78.3 (± 3.2)	6.8 (± 0.1)	1.81 (± 0.07)	3.22 (± 0.09)
45 °	55.8 (± 3.7)	5.7 (± 0.4)	1.69 (± 0.05)	1.91 (± 0.03)
90 °	47.1 (± 2.4)	4.1 (± 0.1)	1.58 (± 0.11)	0.99 (± 0.07)
